# Weathering the storm: how attachment and gender influence coping with racial discrimination

**DOI:** 10.3389/fpsyg.2026.1709353

**Published:** 2026-03-13

**Authors:** Reante Talton, Angel S. Dunbar, Fanita A. Tyrell

**Affiliations:** 1 Department of Psychology, University of Maryland, College Park, MD, United States; 2 Department of African American and Africana Studies, University of Maryland, College Park, MD, United States

**Keywords:** attachment, black youth, coping, discrimination, parent-child relationships

## Abstract

Racial-ethnic microaggressions (REMS) are subtle forms of racial discrimination that negatively affect an individual's mental health. Black individuals often employ diverse coping strategies to navigate these experiences to mitigate the effects of sociocultural stress. However, less research has explored the role of parental influence on the link between REMS and coping strategies. Therefore, the current study examines the association between REMS and collective coping or expressive suppression using parental attachment security as a moderator. Data for this study was drawn from a cross-sectional sample of Black emerging adults (*N* = 230; *M*_*age*_ = 19.99, 69.1% female, 73.9% immigrant-origin background) who attended universities in the Mid-Atlantic region of the United States. Findings revealed that emerging adults with strong maternal bonds were more likely to use adaptive coping responses (i.e., collective coping), suggesting that secure mother–youth attachment can help protect against the negative effects of racial stress. However, low father–youth attachment and low mother–youth attachment strengthened the link between racial-ethnic microaggressions and expressive suppression, suggesting that weak attachment bonds with both fathers and mothers may increase an individual's propensity to suppress their emotions in response to racial discrimination. Cumulatively, these findings suggest that fostering positive parent-youth attachments may be instrumental in helping youth cope effectively with race-based stress.

## Introduction

In the United States of America, structural inequities and systemic barriers have long impacted Black and Brown communities. These inequities do not operate in isolation and are often ingrained in different social institutions and systems. These systems (e.g., education, healthcare, legal) often create an environment where racial discrimination thrives, thus impacting the lives and adjustment of youth from minoritized backgrounds. Consequently, minority youth, especially Black youth often report experiencing high rates of racial discrimination (Pew Research Center, 2024), which is defined as the unequal treatment of persons or groups based on their race or ethnicity (Ahmed et al., 2007). There are various forms of racial discrimination, with one of the most prevalent being racial-ethnic microaggressions (REMS). REMS are subtle, often unintentional, or unconscious forms of racial discrimination that negatively affect the victims' mental health (Nadal et al., 2014a). According to Pew Research Center (2024), 75% of Black individuals report experiencing REMS, with 13% reporting they experience it regularly and 62% reporting they experience it occasionally. The prevalence of these racialized experiences has significant implications for individuals' development, especially for emerging adults who are undergoing changes in their identity development, emotional health, and social lives.

Emerging adulthood, roughly ages 18–25, is a critical period marked by heightened developmental sensitivity. During this time, young adults are more susceptible to emotional distress, mental health challenges, and are especially sensitive to social experiences, particularly those related to identity and belonging (Arnett, 2015). According to Erikson (1968) and Marcia (1966) theory of identity development, identity formation is an ongoing process by which a person comes to understand who they are over time and across different situations. This sense of identity fosters feelings of inner consistency, or “sameness,” that helps individuals feel grounded in themselves, while also allowing for a clear sense of distinction from others (Erikson, 1968; Kerig et al., 2012). In emerging adulthood, there is often a strong emphasis on identity development, particularly as it relates to race, ethnicity, and cultural background. Previous studies (e.g., Branscombe et al., 1999; Pahl and Way, 2006; Sellers and Shelton, 2003) have suggested that experiences of racism often contribute to the identity development process. Specifically, Branscombe et al. (1999) proposed the Rejection–Identification Model (RIM) that emphasizes the process by which members of stigmatized groups psychologically respond to persistent discrimination. The theory shows that although discrimination can negatively impact individuals, it can simultaneously strengthen their identity to their minority group. In other words, experiencing discrimination can lead to a stronger racial or ethnic identity over time. However, research has also shown that experiencing racial discrimination can lead to lasting emotional and psychosocial consequences. For example, research with Black and Latinè college students showed that individuals who experienced frequent racial-ethnic discrimination or microaggressions, reported lower self-esteem (Nadal et al., 2014b) and higher rates of anxiety and depressive symptoms (Nadal et al., 2014a).

## Racial-ethnic microaggressions and coping

Theoretical models, such as the Phenomenological Variant of Ecological Systems Theory (PVEST; Spencer et al., 1997) and the Biopsychosocial Model of Racism (Clark et al., 1999), propose that Black individuals utilized different coping strategies to navigate stressful and challenging environments (Clark et al., 1999). Sanchez et al. (2018) defined coping as cognitive and behavioral efforts that are employed to manage demands that exceed an individual's resources. Coping strategies have generally been categorized into two distinct categories: maladaptive and adaptive. However, recent literature has challenged this binary view, emphasizing that the classification of a coping strategy as adaptative or maladaptive depends on the environmental fit between the stressor and the coping mechanism (Bendezú and Wadsworth, 2017; Tyrell et al., 2024). Notably, in some contexts, coping strategies that have been traditionally labeled as maladaptive may serve protective or functional roles. For example, Black families and caregivers often encourage the use of expressive suppression, the act of inhibiting any outward expression of negative emotions outside the family environment (Gross and Levenson, 1993). Although expressive suppression may be perceived as a maladaptive coping style given its association with heightened internal and physiological arousal (Szczygieł and Maruszewski, 2015), Black families often encouraged its use as a survival strategy to help children navigate racially unsafe environments in the United States of America (Stern et al., 2022). These findings suggest that the adaptative utility of a coping strategy does not only depend on the appraisal of the stressful events but on the individual's personal experiences and social context (McEwen, 1998; Tyrell et al., 2024).

Despite the complexity of research on coping, empirical evidence has continued to associate adaptive coping responses with more favorable developmental outcomes (Spencer et al., 1997). Behaviors such as seeking social support or leaning into community during times of distress (i.e., communal or collective coping) are often considered adaptive coping strategies among Black individuals (Ibe-Lamberts et al., 2025). Prior to the forced migration of Africans due to slavery in the United States of America, strong social networks were an integral component of social organization within the Black community (Kim and McKenry, 1998; Staples and Johnson, 1993). This pattern of strong kinship and community-based networks persisted throughout the enslavement period and continued to evolve in response to the social, economic, and political challenges faced by Black/African American individuals in the United States (Kim and McKenry, 1998). Prior research has shown that collective coping is valued because it is often linked to more positive long-term health outcomes, including improved mental health (Sanchez et al., 2018), greater resilience, and a higher quality of life (Jacob et al., 2023). In contrast, aforementioned coping strategies such as expressive suppression have been considered maladaptive and have been linked to a range of negative outcomes, including heightened psychological distress, struggling to connect with others, and experiencing more distant or strained relationships (Finley et al., 2024). Therefore, while an individual may appear composed on the outside, the internal emotional burden of suppressing their emotions often resides and can even intensify (Dunbar et al., 2017; Gross and Levenson, 1993), leading to negative physical health outcomes over time despite its short-term social protection and benefits.

Nevertheless, both adaptive and maladaptive coping strategies often operate as mechanisms that could explain the linkages between sociocultural stress and health outcomes (Dotterer and James, 2018; Sanchez et al., 2018; Wiggins et al., 2023). Given the importance of coping for adaptation, it is crucial to understand how REMS are linked to different coping strategies, as well as the factors that may influence this association. Historically, researchers examining these associations have focused on the role of racial-ethnic socialization (RES). RES refers to the transmission of both explicit and implicit messages about race and ethnicity from caregivers to their children and includes practices such as cultural socialization, preparation for bias, promotion of mistrust, and egalitarianism (Hughes et al., 2006). Together, these processes aim to equip minoritized children with the skills needed to navigate, combat, and succeed in the face of racial discrimination within society. Previous research has found that RES has consistently served as a protective factor against the negative effects of racial discrimination across several domains including psychological wellbeing (Jiménez and Glover, 2023; Reynolds and Gonzales-Backen, 2017), resilience (Brown and Tylka, 2010), externalizing problems (Henry et al., 2015; Saleem and Lambert, 2016), and academic achievement (Wang and Benner, 2016; Metzger et al., 2020). However, these associations have yet to be examined within other key parenting constructs.

## Attachment security as a resilience factor

Parent-child relationships play a crucial role in the development of effective coping behaviors and responses to stress. Attachment theory has often been used to explain the unique and intricate bond between a parent and their child. In 1969, Bowlby first introduced the theory of attachment as infants instinctively seeking out their caregivers for comfort and reassurance during times of distress (Bowlby, 1969, 1982). This theory emphasizes the importance of a child's confidence in their caregiver's ability to be available and responsive when they need them, especially during times of stress. The stronger the child's confidence and trust in their caregiver's availability and responsiveness to them, the more secure a child feels in the relationship- which in turn reflects the overall quality of the relationship. This sense of security develops through repeated caregiving experiences, particularly experiences that are characterized by consistent and sensitive parenting responses to the child's needs.

These early attachment bonds contribute to the development of internal working models, which are mental representations of the self and others that reflect consistent themes such as trust, emotional availability, and safety (Pietromonaco and Barrett, 2000). Internal working models can either be secure or insecure, depending on the quality of early caregiving experiences. When caregivers are consistent, responsive, and emotionally available, individuals are more likely to develop secure attachments and internal working models (Shaver et al., 1996). In contrast, caregiving that is inconsistent or rejecting often leads to lower attachment security and the formation of insecure internal working models (Shaver et al., 1996). Importantly, while caregiving behaviors and parental sensitivity shape these attachment experiences, attachment itself reflects the child's internalized perception of the relationship rather than specific parenting practices. Therefore, attachment reflects a broader relational context through which parenting behaviors and parental sensitivity may influence children's socioemotional development, as they shape how individuals anticipate others will respond to their emotional needs throughout life.

When attachment theory was originally developed, it was framed largely through the lens of a nuclear-family structure that centered on observations of relational dynamics between a primary caregiver and child in Uganda and in the United States (Ainsworth, 1967). Although this observational method emerged from work conducted within both collectivistic and individualistic cultures, the research at the time did not fully account for other members of the family system as potential secure bases with whom children can form attachment. However, more recent literature, has expanded this perspective through the African proverb “it takes a village to raise a child,” which highlights how children often form multiple attachment relationships with various family members (Reupert et al., 2022). Research conducted in collectivistic cultures such as Uganda (Dagan et al., 2021), China (Xing et al., 2016), as well as in more Individualistic countries like the United States that has other collectivistic-oriented cultural groups such as African Americans (Jackson, 1993), has supported the existence of multiple attachment systems despite the difference in how these relationships may present across cultures. However, these cultural differences do not negate the importance of the attachment relationship with a primary caregiver, whether that caregiver is the mother or father of the child.

Attachment representations formed in early relationships with caregivers lay the groundwork for how individuals approach close relationships throughout life (Sroufe, 2005). While these representations naturally shift over time, they continue to shape expectations around comfort, trust, and how individuals cope into emerging adulthood. Children who have secure attachments in early childhood tend to show higher self-confidence, better self-esteem, and greater emotional regulation later in life (Sroufe, 2005). These attachment patterns are often shaped by both maternal and paternal influences, whose roles may contribute in different ways to a child's development.

Historically, attachment theory has primarily viewed mothers as the primary attachment figure for children, while fathers were often seen more as playmates and disciplinarians than as sources of comfort (Allen et al., 2007; Bretherton, 2010). Research with mothers as the primary caregivers has largely been influenced by cultural and gender norms that shape expectations for women's and men's behavior. Historically, caregiving has been predominantly assigned to women, who have been socially deemed more “nurturing,” while men have not been expected to perform the same duties (Ciaffoni et al., 2025). Specifically, within the context of Black family structures, it is also imperative to highlight the historical implications of caregiving in the U.S. For example, Hill (2001) discusses the disconnect between Western societal expectations of a two-parent nuclear family and the matriarchal system that has historically characterized Black families, a divergence that was not only shaped by slavery but also systemic and economic disparities affecting Black men.

Although culturally embedded gendered norms have devalued the role of men within the caregiving domain, research increasingly suggests that fathers play an equally important, if not more significant, role in shaping children's long-term attachment representations. For example, Grossmann et al. (2002) found that fathers who reported higher levels of attachment security with their children were more likely to have children who developed stronger attachment representations over time. In addition, research has shown that the quality of the father-child relationship and paternal warmth are often associated with fewer externalizing symptoms (Sandler et al., 2008), better emotion regulation skills (Islamiah et al., 2023) and reduced substance use among African American adolescents (Salem et al., 1998). Therefore, youth who receive emotionally supportive guidance from fathers may be less likely to engage in maladaptive coping strategies.

Prior research has shown that maternal behaviors are often linked to internalizing outcomes, such as the development of adaptive emotion regulation skills (Eisenberg et al., 1998). This may be due to the fact mothers are more likely to engage in emotion and coping socialization (i.e., explicit or implicit messages that are conveyed to children about how to cope with stress and their emotions; Morris et al., 2007). For example, when mothers engage in coping behaviors, adolescents are more likely to demonstrate resilience and report fewer internalizing symptoms, even when maternal depression is present (Anderson et al., 2021). Within Black communities, collective coping and social support are especially important strategies, ones that Black mothers often play a central role in fostering. Together, these findings highlight the distinct roles that mothers and fathers play in shaping the development outcomes of their offsprings.

When it comes to coping with racial discrimination specifically, several studies have shown that positive parenting, across both mothers and fathers, can strengthen adolescents' ability to manage stress related to these experiences. For example, empirical evidence indicates that when faced with racial discrimination, youth who reported higher levels of parental warmth and cultural socialization tend to exhibit fewer depressive symptoms (Dotterer and James, 2018) and problem behaviors (Yan et al., 2024). Other studies showed that nurturant parenting (i.e., high levels of parental warmth, inductive reasoning, open communication, and effective child monitoring) can protect youth from the negative effects of racial discrimination (Wiggins et al., 2023). However, few studies have directly examined how parent-child attachment security influences the association between REMS and coping strategies, or whether these parental influences on coping differ based on the child's gender.

## Gender differences

Limited research has explored how the associations among REMS, parent attachment, and coping may differ across same-gender or cross-gender dyads. However, societal gender norms may inform the ways in which mothers and fathers instill coping strategies in their children. Extensive research has shown that parents engage in different emotion socialization processes depending on whether their children present in more feminine or masculine ways (Eschenbeck et al., 2007; Zimmermann and Iwanski, 2014). Specifically, Farrell et al. (2023) found that parents are more likely to use language that encourages emotional expression with their daughters, whereas this type of language is used less frequently with sons. When children are raised in environments devoid of emotional language, they may develop the belief that emotions are unimportant or inappropriate, which eventually can lead to their engagement in emotional suppression. Additionally, parental emotion socialization varies across cultural contexts (Farrell et al., 2023). For Black families in particular, caregivers must often account for the social threats present in the society in which they live, tailoring their children's emotional socialization to reflect the intersection of both gender and racial identity (Crenshaw, 1991). For example, within the Black community, girls are often socialized to engage in more adaptive coping strategies, such as seeking social support, whereas boys are frequently encouraged to internalize and suppress emotional expression (Labella, 2018). For example, Lewis et al. (2013) found that Black college women were more likely to engage in collective coping strategies, while other empirical evidence suggest that Black men tend to rely more on avoidant coping when responding to racism to ensure survival (Sheu and Sedlacek, 2004; Stern et al., 2022).

For other members of the Black diaspora, research shows that Black immigrant youth in the United States face a “triple adaptation task” where they must navigate not only the normative developmental challenges and the structural inequalities that shape life for Black Americans, but they must also deal with the additional psychological and cultural demands associated with being an immigrant (Ferguson et al., 2012; Tyrell et al., 2024; Suárez-Orozco et al., 2018). This unique triple adaptation allows different groups to employ different coping strategies. For example, in an extensive literature review, research has shown that Afro-Caribbean and African immigrants to the U.S. are more likely to engage in more cultural forms of coping (i.e., actively engaging in cultural activities such as dance and music), while African Americans tend to engage in more substance use and activism (Ibe-Lamberts et al., 2025). This intersection of race and gender differences in coping may also be influenced by early attachment experiences.

Researchers have largely focused on the role of parental attachment during childhood. This is because peers and romantic partners become more central as children move through adolescence and into emerging adulthood (Arnett, 2015; Hazan and Shaver, 1994). Although the parent-child attachment relationship may evolve over time, parents still serve as a secure base because the underlying need for emotional closeness, guidance, and stability often remains. There is also evidence to suggest that parental attachment may shape how individuals cope with racial stressors, with some differences emerging based on gender. For example, a study with Black adolescents showed that involved-vigilant parenting (i.e., parenting characterized by inductive reasoning, mutual problem solving, active monitoring, and consistent discipline) was most protective against problem behaviors and racial discrimination when the parent and child shared the same gender, mothers for daughters and fathers for sons (Varner et al., 2021). On the contrary, among cross-gender parent–child pairs, involved-vigilant parenting was related to fewer problem behavior but did not reduce the effects of racial discrimination in the same way it did for same-gender pairs (Varner et al., 2021). This empirical data highlights how important both mothers and fathers are in shaping their offspring coping responses. However, gaps still remain in our understanding of how parent-child dynamics such as attachment security influence coping strategies in the context of REMS.

## Current study

The present study builds on the existing literature to examine how parental attachment security (mothers and fathers) are associated with coping responses to racial-ethnic microaggressions among Black emerging adults. Specifically, we examine whether (1) experiences of racial-ethnic microaggressions (REMS) are associated with collective and expressive suppression coping strategies; (2) mother–youth and father–youth attachment security moderates these associations; and (3) how these effects vary by youth's gender. In alignment with Sanchez et al. (2018) findings that suggest that racial-ethnic microaggressions are positively associated with both adaptive and maladaptive coping strategies, we hypothesized that racial-ethnic microaggressions would be positively associated with both collective coping and expressive suppression. We further hypothesized that parental attachment security would moderate these associations, such that higher attachment security would strengthen the link between racial ethnic microaggressions and collective coping but weaken the link between racial ethnic microaggressions and expressive suppression. Lastly, we explore whether these moderation patterns differ between males and females.

## Method

### Participants

This study utilized data from a cross-sectional study that examined how different sociocultural processes influence Black youth's mental and physiological health. Participants in this study were Black college students who attended universities in the Mid-Atlantic region of the United States. To be eligibility for the study, participants were required to identify as Black/African American, be an undergraduate student, be between the ages of 18 and 25, be fluent in English, and express willingness to have their blood and saliva collected.

The sample was comprised of 230 Black emerging adults (*M*_*age*_ = 19.99, *SD* = 1.68). Most participants identified as female (69.1%) and 73.9% of participants came from an immigrant-origin background (27.1% first generation, 61.8% second generation, and 11.2% third generation). The majority of participants were enrolled at a Predominately White Institution (93.5%), and their standing was distributed as follows: 24.8% freshmen, 24.3% sophomores, 28.7% juniors, and 22.2% seniors. Participants also reported their mothers' education level (4.3% had less than a high school education, 24.8% had completed high school or earned a GED, 3.9% were vocational school graduates, 6.1% held an associate degree, 24.8% had a bachelor's degree, 23.5% had a master's degree, and 10.9% had a professional degree).

### Procedure

Participants were recruited through flyers, emails, campus fair events, social media, and word-of-mouth. After confirming their eligibility through a prescreening survey, participants were then invited to complete a 3 to 3 $\frac{1}{2}$ h in-person lab visit during the morning at either 8 AM or 10 AM as participants were required to give blood samples after a period of fasting. Once consent was given, participants completed a series of self-reported mental health (i.e., stress, coping strategies, parent-child relationship, psychological wellbeing) and demographic questionnaires using the Qualtrics survey platform on an iPad. After the questionnaires were completed, participants completed hand photographs and several physiological assessments, including a dual-energy X-ray absorptiometry (DEXA) scan, a 30 mL blood draw in the laboratory, and saliva collection (4 1.0–1.5 mL samples of passive drool) at home. A small, random subset of individuals also participated in a stress test at the end of the in-person session. Participants were given a break and were allowed to use the restroom whenever needed during the in-person lab session. Participants were also given water throughout the session and at the end of the data collection they were given a snack. All of the study procedures were approved by the Institutional Review Board (IRB protocol #: 1853726-17).

### Measures

#### Self-report questionnaires

##### Racial and ethnic microaggressions

Racial-ethnic microaggressions was assessed using the racial and ethnic microaggressions scale (REMS; Nadal, 2011). The REMS is a 45-item self-report questionnaire that is designed to assess the frequency of the unconscious and daily experiences of racial discrimination by marginalized communities. The scale uses a dichotomous response scale ranging from 0 (*I did not experience this event*) to 1 (*I experienced this event at least once in the past six months*). The scale captures experiences of microaggressions across five subscales, including the Second-Class Citizen and Assumptions of Criminality subscale (6 items), which was used to assess secondary class citizen assumption (i.e., experiences in which individuals of color experience substandard treatment compared to White individuals; α = 0.89). Sample item on this REMS subscale includes “I was ignored at school or work because of my race.” Higher scores reflect more frequent experiences of REMS.

##### Parent-youth attachment security

Parent–youth attachment security was assessed using the Inventory of Parent and Peer Attachment (IPPA; Armsden and Greenberg, 1987). The IPPA is a 9-item measure that capture youth's perceptions of the positive and negative affective/cognitive dimensions of their relationships with mothers (i.e., mother–youth attachment [MYA], α = 0.94) and fathers (i.e., father–youth attachment [FYA], α = 0.95). This measure is comprised of two subscales including: Trust (e.g., “I trusted my mother/father”) and Communication (e.g., “My mother/father helped me talk about my problems and difficulties”). The responses to this scale are measured on a 5-point Likert scale ranging from 1 (*almost never or never true*) to 5 (*almost always or always true*). For the IPPA scale, higher scores reflect higher levels of parent–child attachment security.

##### Expressive suppression

The Emotion Regulation Questionnaire (ERQ; Gross and John, 2003) is a 10-item scale that measures an individual's emotion regulation strategies. One of the two subscales assesses expressive suppression (i.e., a form of response modulation that involves inhibiting ongoing emotion–expressive behavior, 4 items, α = 0.70). The responses to this scale are on a 7-point Likert scale ranging from 1 (strongly disagree) to 7 (strongly agree). A sample item includes “When I am feeling negative emotions, I make sure not to express them.” Higher scores on the subscale reflect a greater use of emotion suppression.

##### Collective coping

Collective coping was assessed using the Africultural Coping Systems Inventory Scale (ACSI; Utsey et al., 2000). ACSI is a 31-item scale that is designed to assess culturally relevant coping strategies that are specific to individuals of African descent. The responses to this scale are on a 4-point Likert-type scale ranging from 0 (*did not use*) to 3 (*used a great deal*). There are four subscales in the ACSI scale, which includes collective coping (i.e., an Afrocentric cultural value in which individuals rely on group-centered activities to cope with stressful situations, 8 items, α = 0.82). A sample item on this subscale includes “Sought emotional support from family and friends.” Higher scores reflect a greater tendency to seek support from one's community in times of stress.

#### Data analytic approach

SPSS 31.0 (IBM Corp, 2020) and *MPlus* 8.1 (Muthén and Muthén, 2017) were used for data analyses. SPSS was used to conduct descriptive and correlation analyses. Path analyses were performed in Mplus with full information maximum likelihood and robust standard errors to address missing data and violations of normality. Path models evaluated the direct effect of REMS on coping and the moderating influences of parent–youth attachment and gender on these associations. In all path models, covariates were included: age, maternal education, gender, and immigration status. Age was included because research has shown that older individuals tend to engage in less maladaptive and more mature coping strategies (Folkman et al., 1987; Segal et al., 2007). Maternal education was also included as a proxy for socioeconomic status because children who are from high income families or have parents who are highly educated tend to express fewer negative emotions (Cheng et al., 2018). Additionally, mothers have historically been deemed as more of the socializing agent that provides a stimulating environment for the child (Farah et al., 2008; Farrell et al., 2023). A correlation between maternal and paternal education was also conducted, and the results indicated a significant positive association (*r* = 0.55, *p* < 0.001). Given concerns about multicollinearity and data missingness (i.e., more data available on mother's education level than father's), maternal education was included in the models. Gender was included as a covariate because, as noted previously, boys and girls are socialized to employ different coping strategies (Farrell et al., 2023). For the gender variable, males were recoded as zero, females were recoded as one, and gender non-conforming individuals were reported as missing. Lastly, immigration status has also been shown to influence coping processes (Ibe-Lamberts et al., 2025). For the immigration status variable, non-immigrants were coded as a zero and the immigrant participants recoded as a one.

A power analysis was conducted using G^*^Power version 3.1.9.7 (Faul et al., 2007) to determine the minimum sample size required to test the study hypothesis. The analysis indicated that a total sample size of *N* = 114 was needed to achieve 80% power to detect a medium effect size at an alpha level of 0.05, specifically for testing 3 covariates (age, maternal education, immigration status), 3 predictors/moderators (REMS, parent–youth attachment (mothers or fathers), gender) and 3 interaction terms. A smaller sample size of *N* = 103 was needed for one moderator. Consistent with our power analysis, primary analyses focused on a single outcome variable. However, additional models, including both outcomes, are reported in the Supplementary material.

To address the research questions, a series of path analyses were conducted, first, a baseline model with all the covariates and REMS, and no moderators were regressed on a single coping outcome (collective coping or expressive suppression). In all models, non-significant covariances were constrained to zero. Building on the baseline model and to test the moderating effect of parental attachment, interaction terms for mother-child attachment or father-child attachment were included in the path models. Simple slope analyses were performed to examine significant interactions by testing the moderator at one standard deviation above and below the mean (Preacher et al., 2007). Finally, to test the moderating effect of gender (male vs. female), we ran a multigroup analysis and tested whether specific interaction terms differ across gender using the Wald Test (Chou and Bentler, 1990). A non-significant Wald Test indicated no group differences and a significant Wald Test indicated group differences.

Model fit was assessed using several indices, including the non-significant chi-square test (Satorra, 2000), Tucker Lewis Index (TLI > 0.95; Tucker and Lewis, 1973) comparative fit index (CFI > 0.95; Bentler, 1990), root mean square error of approximation (RMSEA < 0.05; MacCallum et al., 1996), and standardized root mean square residual (SRMR < 0.08; Hu and Bentler, 1999). In the models, initial estimation of all paths revealed several non-significant associations. These non-significant paths were constrained across groups to improve model parsimony. These non-significant paths did not substantially alter the overall pattern of the results or model fit. Therefore, the model fit indices reported in the result section are based on parsimonious models that include constrained parameters (e.g., the covariance between maternal education with age was fixed at zero).

## Results

### Descriptive statistics and zero-order correlations

Descriptive statistics and zero-order correlations for the study variables are presented in Table 1. Zero-order correlations revealed that REMS was not significantly correlated with either collective coping or expressive suppression. Similarly, REMS was not related to maternal-youth or paternal-youth attachment security. However, when examining the association between maternal attachment security and coping strategies, mother-youth attachment had a positive significant association with collective coping and a negative significant association with expressive suppression. For paternal attachment security, there was a positive and significant association with collective coping, but there was no significant association with expressive suppression.

**Table 1:** Descriptive statistics and zero order correlations for study variables.

**Variable**	***n***	***M***	***SD***	**1**	**2**	**3**	**4**	**5**	**6**	**7**	**8**	**9**
1. REMS	228	10.23	5.47	—	—	—	—	—	—	—	—	—
2. CC	224	7.83	5.25	0.09	—	—	—	—	—	—	—	—
3. ES	224	15.90	5.02	−0.11	−0.01	—	—	—	—	—	—	—
4. MYA	224	29.78	9.97	−0.07	0.19^**^	−0.16^*^	—	—	—	—	—	—
5. FYA	205	25.44	10.63	−0.10	0.18^**^	−0.09	0.38^**^	—	—	—	—	—
6. Age	229	19.95	1.57	0.09	0.16^*^	0.02	0.03	0.01	—	—	—	—
7. Gender	220	0.72	0.45	−0.18^**^	0.09	−0.23^**^	−0.02	−0.05	−0.21	—	—	—
8. Maternal Education	226	4.37	1.88	0.05	0.02	−0.11	0.15^*^	0.01	0.03	−0.02	—	—
9. Immigration Status	230	0.74	0.44	−0.01	−0.08	0.04	−0.13	−0.11	−0.08	−0.11	0.05	—

^*^p < 0.05.

^**^p < 0.01.

^***^p < 0.001.

^†^Marginally significant effect (0.05 ≤ *p* < 0.10).

REMS, racial-ethnic microaggressions; MYA, mother-youth attachment; FYA, father-youth attachment; CC, collective coping; ES, expressive suppression; M, mean; SD, standard deviation; Gender was coded as 0 = male, 1 = female; Immigration status was coded as 0 = no immigrant background, 1 = immigrant background; n = Sample Size.

### Aim 1: The direct effects between REMS and coping strategies

Two direct path models were conducted to evaluate the influence of racial-ethnic microaggressions on collective coping and expressive suppression (see Table 2). The model fit for collective coping was acceptable, *X*^2^ = 5.019, *df* = 7, *p* = 0.6577; *RMSEA* = 0.000, 90% [0.000, 0.065]; *CFI/TLI* = 1.000/1.000; *SRMR* = 0.028. REMS were not associated significantly with collective coping, *t* = 1.364, *p* = 0.172. Age, *t* = 2.600, *p* = 0.009, and female gender, *t* = 2.004, *p* = 0.045, were significant and positively associated with collective coping, but maternal education, *t* = −0.353, *p* = 0.724, and immigration status, *t* = −0.671, *p* = 0.502, were not.

**Table 2:** The direct effects of racial-ethnic microaggressions on collective coping and expressive suppression in black emerging adults.

**Predictors**	Collective coping		Expressive suppression	
	β	***SE***	β	***SE***
REMS	0.10	0.07	0.08	0.06
Age	0.18^*^	0.07	−0.03	0.07
Gender	0.13^*^	0.07	−0.22^**^	0.07
Maternal education	−0.03	0.07	−0.11^†^	0.07
Immigration status	−0.05	0.07	0.02	0.07
*R* ^2^	0.05^†^	0.03	0.07^*^	0.04

^*^p < 0.05.

^**^p < 0.01.

^***^p < 0.001.

^†^Marginally significant effect (0.05 ≤ *p* < 0.10).

REMS, racial-ethnic microaggressions; SE, standard error; Gender was coded as 0 = male, 1 = female; Immigration status was coded as 0 = no immigrant background, 1 = immigrant background; β = standardized regression coefficient.

For expressive suppression, the model fit was acceptable, *X*^2^ = 4.951, *df* = 7, *p* = 0.6660; *RMSEA* = 0.000, 90% [0.000, 0.065]; *CFI/TLI* = 1.000/1.000; *SRMR* = 0.028. REMS were not associated significantly with expressive suppression, *t* = 1.345, *p* = 0.178. Female gender (*t* = 3.330, *p* = 0.001) was negatively associated with expressive suppression, but age (*t* = −0.425, *p* = 0.671), maternal education (*t* = −1.652, *p* = 0.098), and immigration status (*t* = 0.328, *p* = 0.743) were not associated significantly with expression suppression.

### Aim 2: The moderating effects of parental attachment on REMS and coping

A series of path analyses were conducted to assess the interaction effects of mother–youth and father–youth attachment on the associations between REMS and coping.

#### REMS, MYA, and collective coping

**Figure 1:**
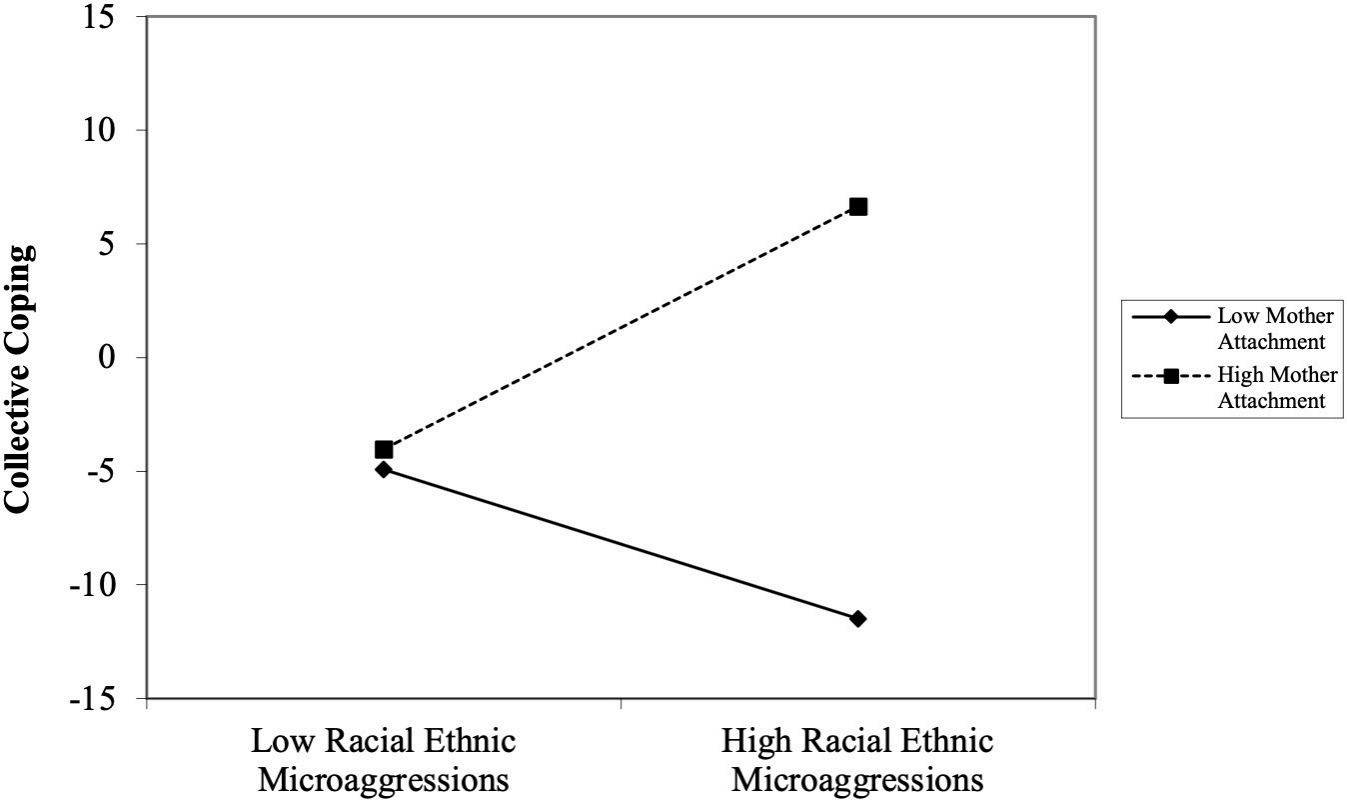
The interaction effect of racial-ethnic microaggressions and mother-youth attachment predicting collective coping among Black emerging adults.

The first path analysis evaluated the moderating effect on mother–youth on the association between REMS and collective coping (see Table 3). The model fit for collective coping was acceptable, *X*^2^ = 4.989, *df* = 7, *p* = 0.6613; *RMSEA* = 0.000, 90% [0.000, 0.065]; *CFI/TLI* = 1.000/1.000; *SRMR* = 0.022. In contrast to the direct effects models, after controlling for the covariates and adding mother–youth attachment to the model, REMS became positively associated with collective coping, *t* = 2.175, *p* = 0.030. Mother-youth attachment, *t* = 3.150, *p* = 0.002, was positively associated with collective coping. Additionally, the interaction between mother–youth attachment and REMS was significant in predicting collective coping, *t* = 2.383, *p* = 0.017. Simple slope analysis further revealed that Black emerging adults who reported experiencing high levels of REMS and high levels of mother–youth attachment were more likely to utilize more collective coping, *b* = 0.502, *SE* = 0.187, *t* = 2.684, *p* = 0.007 (see Figure 1). These effects were not evident among emerging adults who reported low levels of mother–youth attachment. *b* = 0.064, *SE* = 0.069, *t* = 0.935, *p* = 0.350.

**Table 3:** Moderated effects of mother-youth attachment and father-youth attachment on the associations of racial-ethnic microaggressions on collective coping and expressive suppression.

**Predictors**	Collective coping		Expressive suppression	
	β	***SE***	β	***SE***
**Mother-youth attachment**				
REMS	0.16^*^	0.07	0.04	0.06
MYA	0.21^**^	0.07	−0.14^*^	0.07
REMS ^*^ MYA	0.17^*^	0.07	−0.12^*^	0.06
Age	0.17^*^	0.07	−0.03	0.07
Gender	0.13^*^	0.06	−0.22^**^	0.07
Maternal education	−0.06	0.07	−0.10	0.07
Immigration status	−0.02	0.07	0.00	0.07
*R* ^2^	0.12^**^	0.04	0.10^**^	0.04
**Father-youth attachment**				
REMS	0.15^*^	0.08	0.00	0.06
FYA	0.20^**^	0.07	−0.10	0.07
REMS ^*^ FYA	0.08	0.08	−0.17^*^	0.07
Age	0.18^**^	0.07	−0.03	0.07
Gender	0.13^*^	0.07	−0.20^**^	0.07
Maternal education	−0.03	0.07	−0.10	0.07
Immigration status	−0.03	0.07	0.01	0.07
*R* ^2^	0.10^*^	0.04	0.10^**^	0.04

^*^p < 0.05.

^**^p < 0.01.

^***^p < 0.001.

REMS, racial-ethnic microaggressions; MYA, mother-youth attachment; FYA, father-youth attachment; SE, standard error; *R*^2^, R-Squared; Gender was coded as 0 = male, 1 = female; Immigration status was coded as 0 = no immigrant background, 1 = immigrant background.

#### REMS, FYA, and collective coping

The second path analysis evaluated the moderating effect of father–youth attachment on the association between REMS and collective coping. The model fit for collective coping was acceptable *X*^2^ = 5.018, *df* = 7, *p* = 0.6577; *RMSEA* = 0.000, 90% [0.000, 0.065]; *CFI/TLI* = 1.000/1.000; *SRMR* = 0.023. Consistent with the previous model, REMS became positively associated with collective coping, *t* = 1.985, *p* = 0.047 after controlling for the covariates and father–youth attachment (see Table 3). Additionally, father–youth attachment was positively associated with collective coping, *t* = 2.901, *p* = 0.004. However, there was no significant interaction between REMS and father–youth attachment on collective coping, *t* = 1.087, *p* = 0.277.

#### REMS, MYA, and expressive suppression

**Figure 2:**
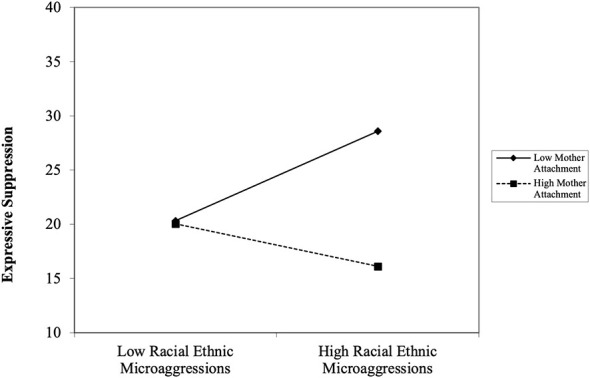
The interaction effect of racial-ethnic microaggressions and mother-youth attachment predicting expressive suppression among Black emerging adults.

The third path analysis evaluated the moderating role of mother–youth attachment on the association between REMS and expressive suppression. The model fit for collective coping was acceptable, *X*^2^ = 4.930, *df* = 7, *p* = 0.6686; *RMSEA* = 0.000, 90% [0.000, 0.065]; *CFI/TLI* = 1.000/1.000; *SRMR* = 0.022. REMS showed no significant association with expressive suppression in this model, *t* = 0.588, *p* = 0.557. Mother-youth attachment was associated negatively with expressive suppression, *t* = −1.983, *p* = 0.047. Additionally, the interaction between mother–youth attachment and REMS was significant when predicting expressive suppression, *t* = −2.089, *p* = 0.037. The simple slope analysis revealed that Black emerging adults who reported experiencing high levels of REMS and low levels of mother–youth attachment were more likely to engage in expressive suppression, *b* = 0.148, *SE* = 0.070, *t* = 2.179, *p* = 0.029 (see Figure 2). These effects were not evident among emerging adults who reported high levels of mother–youth attachment, *b* = −0.085, *SE* = 0.090, *t* = −0.998, *p* = 0.318.

#### REMS, FYA, and expressive suppression

**Figure 3:**
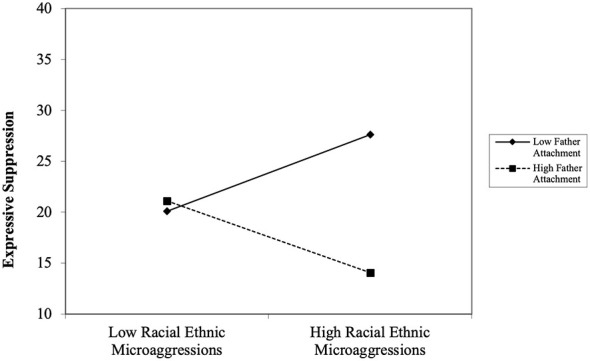
The interaction effect of racial-ethnic microaggressions and father-youth attachment predicting expressive suppression among Black emerging adults.

The fourth path analysis evaluated the moderating role of father–youth attachment on the association between REMS and expressive suppression. The model fit for collective coping was acceptable, *X*^2^ = 4.965, *df* = 7, *p* = 0.6642; *RMSEA* = 0.000, 90% [0.000, 0.065]; *CFI/TLI* = 1.000/1.000; *SRMR* = 0.022. REMS showed no significant association with expressive suppression, *t* = 0.050, *p* = 0.960 in this model. Father-youth attachment was not significantly related to expressive suppression, *t* = −1.455, *p* = 0.146. However, the interaction between father–youth attachment and REMS was significant in predicting expressive suppression, *t* = −2.683, *p* = 0.007). The simple slope analysis revealed that Black emerging adults who reported experiencing high levels of REMS and low levels of father–youth attachment were more likely to use expressive suppression, *b* = 0.154, *SE* = 0.070, *t* = 2.315, *p* = 0.021 (see Figure 3). At high levels of father–youth attachment, these effects showed a marginal negative trend between REMS and expressive suppression, *b* = −0.149, *SE* = 0.090, *t* = −1.682, *p* = 0.092.

### Aim 3: The moderating effects of gender and attachment on REMS and coping

A series of Wald chi-square tests were performed to evaluate the moderating effect of gender on the associations among REMS, mother–youth attachment or father–youth attachment and collective coping or expressive suppression. Across all models, the Wald test analyses [Wald χ^2^_(1)_ = 0.152–1.411, *ps* = 0.2350–0.6963] revealed no significant differences across gender.

Additional models (direct and moderation) were estimated with both outcomes included in the same model. These models did not yield significant interaction results (see Supplementary material), potentially indicating insufficient power to estimate models that include both outcomes. In addition, several models were conducted to test the moderating effect of immigration status instead of gender on the associations among REMS, mother–youth attachment or father–youth attachment and collective coping or expressive suppression; however, these analyses also yielded non-significant results (see Supplementary material).

## Discussion

Black emerging adults in the United States carry the burden of navigating multiple sociocultural stressors, including racial and ethnic microaggressions, and often engage in an array of coping strategies to mitigate their effects. While the association between racial-ethnic discrimination and coping has been widely explored (Clark et al., 1999; Gaylord-Harden and Cunningham, 2009; Sanchez et al., 2018; Seaton et al., 2014), little is known about the role of parental attachment (both mother–youth attachment and father–youth attachment) on this association. Thus, this study explored the association between racial-ethnic microaggressions and coping strategies and evaluated whether these associations differed by parental attachment. Overall, the findings revealed that mother–youth attachment security was a significant moderator on the association between racial-ethnic microaggressions and collective coping while both mother–youth and father–youth attachment security were significant moderators of the association between racial-ethnic microaggressions and expressive suppression.

### Racial-ethnic microaggressions and coping strategies

Hypothesize 1 was partially supported. Racial-ethnic microaggressions showed no significant relationship with collective coping and expressive suppression in the direct models. However, racial-ethnic microaggressions were positively associated with collective coping but not expressive suppression after controlling for age, gender, maternal education, immigration status, and parent-youth attachment. According to these findings, emerging adults who were exposed to high levels of racial discrimination were more likely to seek support and resources from their community when experiencing racial distress. Collective coping is often seen as an adaptive form of cultural coping that is grounded in Afrocentric values (i.e., valuing community support over individual reliance; Jacob et al., 2023), whereas expressive suppression (i.e., inhibiting one's emotions when dealing with stress) is considered a maladaptive coping because it leads to internal physiological arousal (Gross and Levenson, 1993). Although previous literature (Sanchez et al., 2018) suggests that individuals who experienced discriminatory stress tend to use both adaptive and maladaptive coping strategies, these findings suggest that Black emerging adults are more likely to use adaptive coping strategies when they experience race-based stress.

Certain individuals were more likely to use collective coping or expressive suppression. Older Black emerging adults and females were more likely to engage in collective coping when compared to younger individuals and males. One possible explanation is that, as individuals age, they tend to develop and rely on healthier coping mechanisms (Brown et al., 1986). As for gender, females may be more likely to engage in collective coping strategies compared to their male counterparts, because young Black girls are often socialized to seek out more social forms of support such as reaching out to family and joining religious groups in times of distress (Lewis-Coles and Constantine, 2006). In contrast, males were more likely to suppress their emotions in response to racial stress. These findings are consistent with empirical data showing that boys are often socialized not to show any emotions, vulnerability, or discuss their feelings (Eschenbeck et al., 2007; Zimmermann and Iwanski, 2014). In the context of the Black or African American community, there is a familiar aphorism that “boys don't cry,” (Labella, 2018; Lindsey et al., 2017). It highlights a stigmatized socialization pattern in child-rearing, in which boys are discouraged from expressing emotions as openly as girls.

### Racial-ethnic microaggressions, parent-youth attachment, and coping strategies

Consistent with our second hypothesis, mother–youth attachment, but not father–youth attachment moderated the association between racial-ethnic microaggressions and collective coping. Specifically, Black emerging adults who experienced higher levels of racial-ethnic microaggressions and reported high levels of maternal attachment security were more likely to engage in collective coping strategies compared to individuals who experienced low levels of racial-ethnic microaggressions. However, similar patterns did not emerge among individuals who reported low levels of maternal attachment security. This finding suggests that mother–youth attachment may function as a protective factor for Black emerging adults because it may buffer against the negative effects of racial-based stress and increases the likelihood that youth will engage in adaptive coping responses.

Notably, attachment security in the current study was assessed based on the degree of mutual trust and quality of communication between parents and their offspring. Previous research suggests that strong attachment bonds begin to form early in life through effective communication between caregivers and their children (Ishak et al., 2010). This early onset of communication lays the foundation for developing trust in caregivers who are available and responsive to the child's needs. When children establish these secure and supportive relationships with their mothers, they learn to rely on them during times of stress. Over time, individuals transfer these internal working models of trust and communication to other relationships, learning to seek support from their communities and significant others when they are stressed (Compas et al., 1988; Freitas and Downey, 1998; Skinner and Edge, 1998).

In contrast to our hypothesis, father–youth attachment did not moderate the association between REMS and collective coping. However, both mother–youth and father–youth attachment emerged as a significant predictor of collective coping in the model. Specifically, Black emerging adults who reported higher levels of mother–youth and father–youth attachment were more likely to rely on their communities for support in times of stress. This finding suggests that stronger attachment to both mothers and fathers may play a meaningful role in promoting collective or adaptive coping strategies in their children.

In contrast to collective coping, both mother–youth attachment and father–youth attachment moderated the association between racial-ethnic microaggressions and expressive suppression. Specifically, our findings revealed that when emerging adults experienced high levels of racial-ethnic microaggressions and low levels of mother–youth or father–youth attachment, they were more likely to suppress their emotions. Interestingly, this positive link was not evident among individuals who reported high levels of mother–youth attachment. At trend level, the data also showed that Black emerging adults who reported high levels of father–youth attachment and experienced high levels of racial-ethnic microaggressions were less likely to suppress their emotions. Cumulatively, the findings suggest that weak mother–youth attachment or father–youth attachment bonds may increase the likelihood that an individual will suppress their emotions or use maladaptive coping strategies to deal with racial discrimination. Moreover, the findings suggest that early attachment relationships have lasting effects on a person's emotional development (Dong, 2024). Considering that trust and communication are key components of attachment security, individuals with more secure attachments to their parents may feel more comfortable sharing and expressing their emotions with others. With similar patterns observed for mother–youth and father–youth attachment, these finding also indicated that both parents play an equally important role in helping youth cope effectively with sociocultural stress.

### Racial-ethnic microaggressions, parent-youth attachment and coping strategies: Moderation by youth's gender

Contrary to our exploratory hypothesis, youth gender did not moderate the associations among racial-ethnic microaggressions, mother–youth attachment or father–youth attachment, and collective coping or expressive suppression. The non-significant findings may suggest that the patterns of association are similar among Black emerging adults regardless of youth gender. However, the lack of significance could also be attributed to the sample size. Detecting interaction effects require larger sample sizes (Shieh, 2010). The smaller sample size may have reduced the statistical power to detect small interaction effects. Therefore, future research should use a larger sample size to replicate these findings.

### Strengths and limitations

This study has several strengths. First, to our knowledge, this study is the first to examine the association between racial-ethnic microaggressions, collective coping, and expressive suppression, as well as the moderating role of parent-youth attachment (both mothers and fathers). Research on attachment security usually focuses on maternal-child attachment (Pederson et al., 1998), even though empirical evidence indicates that the influence of fathering on child development increases as individuals age. This study adds to the scant literature on father–youth attachment. In addition, the inclusion of parental attachment into the discussion of racial stress and health outcomes, contributes to the overall field by broadening our understanding of the relational dynamics that influence coping processes in emerging adults. Finally, this study examined parent-youth attachment in a sample that consisted of only emerging adults (i.e., individuals between ages 18–25). Parents are often overlooked during this stage of development; however, these findings suggest that parents still serve as a secure base during this transition despite changes in the individual's social network and resources (Hazan and Shaver, 1994; Arnett, 2015).

Another strength of this study is its diverse sample. The participants were predominantly Black individuals of immigrant origin, with a small subsample of African Americans. Thus, this sample represents a population that is often underrepresented in psychological research. Moreover, the majority of the sample included individuals from moderate to high socioeconomic backgrounds. Research with Black individuals is usually comprised of individuals from low-income backgrounds (Perry et al., 2024; Bloome, 2014; Collins et al., 2000). However, using a sample with Black individuals from more diverse socioeconomic backgrounds offers a more nuanced perspective about the processes that support positive development and adaptation in this population beyond the high-risk or low-income groups commonly studied.

Although this study had several strengths, it was not without its limitations—which may be integral to how the findings are interpreted. First, this study predominantly relied on youth self-report. Using a single reporter may lead to shared method bias or may omit different perspectives from the parents (i.e., parent–youth attachment) that could be important to understand these associations. However, research has suggested that parents are more susceptible to over-reporting their child's positive behaviors (Abar et al., 2015) and their own positive parenting behavior (Morsbach and Prinz, 2006), so youth self-report may still be valuable. Future studies examining these associations should incorporate reports from multiple informants to reduce bias and gain a more holistic understanding of the parent–child dynamics. Second, the cross-sectional nature of this study limits interpretations regarding the direction of effects. Future researchers should use longitudinal data to determine the direction, reciprocal and transactional associations among racial-ethnic microaggressions, parental attachment, and coping. Third, this study used only one of the five racial/ethnic microaggression subscales, which may have limited our holistic understanding of participants' experiences with racial-ethnic microaggressions. Future research should use the full measure or other subscales to determine whether similar or different patterns of findings will emerge. Fifth, while attachment research has traditionally focused on individualistic nuclear family systems, recent work increasingly examines families with multiple attachment figures, particularly in collectivistic cultures, and the ways these figures interact (Bacro et al., 2021; Crittenden and Dallos, 2009; Dagan et al., 2021). Future research should examine how children from diverse cultures with attachments to multiple figures within a family structure cope with stressors, and how these attachment systems go on to influence subsequent socioemotional developmental outcomes. This will not only improve the generalizability of findings but also deepen our understanding of how attachment processes operate across different cultural and familial contexts. Finally, this study did not examine racial-ethnic socialization as a predictor of youth coping strategies, nor did it explore how ethnic vs. racial identity may shape developmental outcomes. Although an overwhelming amount of literature has examined the moderating role of racial-ethnic socialization and identity on the association between racial discrimination and multiple developmental outcomes (Caughy et al., 2006; Huguley et al., 2019; Nelson et al., 2018; Wang et al., 2020), future research should investigate these processes to better understand how identity and socialization jointly influence Black emerging adults' coping and developmental outcomes, especially in immigrant populations where ethnic identity may be more salient (Agi and Rivas-Drake, 2022).

### Implications

The findings from this study offer several important implications for both research and clinical practice. First, the results suggest that fostering positive mother–youth and father–youth attachment might be instrumental in helping emerging adults cope with sociocultural stressors such as racial-ethnic microaggressions. Clinicians can further support the development of Black emerging adults by developing interventions aimed at strengthening parent–child relationships and promoting adaptive coping strategies in racially marginalized youth. Additionally, teachers and practitioners working with Black and immigrant-origin families may consider incorporating culturally sensitive approaches into their work that could engage parents in discussions around emotional expression, coping, and racial socialization. Considering that father–youth attachment was a significant protective factor against racial discrimination, clinicians may want to consider developing interventions that include Black fathers, focusing on ways to support positive coping skills and emotional development in their daughters and sons.

The gender differences observed in this study suggest that boys and girls may experience and respond to sociocultural stress differently. This highlights a need for gender-responsive strategies in both research and intervention efforts. In particular, since boys tend to suppress their emotions, clinicians should consider developing interventions for parents that teach them the importance of encouraging appropriate emotional expression for all youth.

This study included a unique participant pool, with the majority of participants being of immigrant background and economically affluent. However, this study did not examine generational immigration status, as the participant pool also included African Americans, which limits our understanding of the nuances within this ethnic group. Future researchers should explore these associations further to investigate how ethnic and racial identity may influence these developmental processes in Black immigrant-origin emerging adults.

Finally, these findings contribute to a growing body of work emphasizing the unique experiences of Black immigrant-origin populations that are often overlooked in psychological research. By focusing on the developmental period of emerging adulthood, this study also underscores the importance of clinicians adopting more inclusive practices that support the use of multiple coping strategies to navigate diverse stressors. Moreover, the findings have important implications for college-based support services, community-based interventions, and parenting programs, particularly within diverse and marginalized communities. Considering the rising racial tensions in the United States, it is critical that researchers and clinicians identify protective factors that can help buffer against the impact of race-based stress and discrimination on youth development.

## Conclusion

Black emerging adults are often exposed to experiences of racial discrimination and inequality. Much of the research in this area has examined the link between racial discrimination and health outcomes, using various coping strategies as a moderator of that association. However, less research has explored the direct linkage between racial discrimination and coping strategies and the potential protective effect of parental attachment on youth adaptation. Specifically, mother–youth attachment was a protective factor on the association between racial-ethnic microaggressions and collective coping. This suggests that strong maternal bonds can mitigate the adverse effects of racial stress. However, low mother–youth and father–youth attachment strengthens the link between racial-ethnic microaggressions and expressive suppression, suggesting that having poor maternal and paternal bonds may increase an individual's propensity to engage in more maladaptive coping strategies when dealing with racial stressors. Future studies should consider other sociocultural processes (e.g., racial-ethnic socialization) to understand what other factors may contribute to effective coping responses and better health outcomes among Black emerging adults.

## Data Availability

The original contributions presented in the study are publicly available. This data can be found here: https://osf.io/k8hmd.

## References

[B1] Abar C. C. Jackson K. M. Colby S. M. Barnett N. P. (2015). Parent–child discrepancies in reports of parental monitoring and their relationship to adolescent alcohol-related behaviors. J. Youth Adolesc. 44, 1688–1701. doi: 10.1007/s10964-014-0143-6 24964878 PMC4511723

[B2] Agi A. C. Rivas-Drake D. (2022). “Other people's battles”? Incorporating the experiences of immigrant-origin Black american youth in ethnic/racial identity research. Identity 22, 101–115. doi: 10.1080/15283488.2021.1919518

[B3] Ahmed A. T. Mohammed S. A. Williams D. R. (2007). Racial discrimination and health: pathways and evidence. Indian J. Med. Res. 126, 318–327. 18032807

[B4] Ainsworth M. D. S. (1967). Infancy in Uganda: Infant Care and the Growth of Love. Baltimore, MD: Johns Hopkins Press.

[B5] Allen J. P. Porter M. McFarland C. McElhaney K. B. Marsh P. (2007). The relation of attachment security to adolescents' paternal and peer relationships, depression, and externalizing behavior. Child Dev. 78, 1222–1239. doi: 10.1111/j.1467-8624.2007.01062.x 17650135 PMC2413435

[B6] Anderson A. S. Watson K. H. Reising M. M. Dunbar J. P. Bettis A. H. Gruhn M. A. . (2021). Relations between maternal coping socialization, adolescents' coping, and symptoms of anxiety and depression. J. Child Family Stud. 30, 663–675. doi: 10.1007/s10826-020-01879-y 40895643 PMC12393037

[B7] Armsden G. C. Greenberg M. T. (1987). The inventory of parent and peer attachment: individual differences and their relationship to psychological well-being in adolescence. J. Youth Adolesc. 16, 427–454. doi: 10.1007/BF02202939 24277469

[B8] Arnett J. J. (2015). “Identity development from adolescence to emerging adulthood: what we know and (especially) don't know,” in The Oxford Handbook of Identity Development (Oxford: Oxford University Press), 53–64.

[B9] Bacro F. Forslund T. Granqvist P. (2021). “Children's multiple attachment relationships and representations in different family contexts,” in Attachment Theory and Research: A Reader (Hoboken, NJ: John Wiley & Sons, Inc.).

[B10] Bendezú J. J. Wadsworth M. E. (2017). If the coping fits, use it: preadolescent recent stress exposure differentially predicts post-TSST salivary cortisol recovery. Dev. Psychobiol. 59, 848–862. doi: 10.1002/dev.21542 28742218

[B11] Bentler P. M. (1990). Comparative fit indexes in structural models. Psychol. Bull. 107, 238–246. doi: 10.1037/0033-2909.107.2.238 2320703

[B12] Bloome D. (2014). Racial inequality trends and the intergenerational persistence of income and family structure. Am. Sociol. Rev. 79, 1196–1225. doi: 10.1177/0003122414554947 26456973 PMC4598060

[B13] Bowlby J. (1969). Attachment and Loss, Vol. 1: Attachment. Attachment and Loss. New York, NY: Basic Books.

[B14] Bowlby J. (1982). Attachment and Loss. Vol. I: Attachment, 2nd edn. New York: Basic (Original work published 1969).

[B15] Branscombe N. R. Schmitt M. T. Harvey R. D. (1999). Perceiving pervasive discrimination among African Americans: implications for group identification and well-being. J. Pers. Soc. Psychol. 77, 135–149. doi: 10.1037/0022-3514.77.1.135

[B16] Bretherton I. (2010). Fathers in attachment theory and research: a review. Early Child Dev. Care 180, 9–23. doi: 10.1080/03004430903414661

[B17] Brown D. L. Tylka T. L. (2010). Racial discrimination and resilience in African American young adults: examining racial socialization as a moderator. J. Black Psychol. 37, 259–285. doi: 10.1177/0095798410390689

[B18] Brown J. M. O' Keefe J. Sanders S. H. Baker B. (1986). Developmental changes in children's cognition to stressful and painful situations. J. Pediatr. Psychol. 11, 343–357. doi: 10.1093/jpepsy/11.3.343 3772682

[B19] Caughy M. O. Nettles S. M. O'Campo P. J. Lohrfink K. F. (2006). Neighborhood matters: racial socialization of African American children. Child Dev. 77, 1220–1236. doi: 10.1111/j.1467-8624.2006.00930.x 16999794

[B20] Cheng F. Wang Y. Zhao J. Wu X. (2018). Mothers‘ negative emotional expression and preschoolers' negative emotional regulation strategies in Beijing, China: the moderating effect of maternal educational attainment. Child Abuse Neglect 84, 74–81. doi: 10.1016/j.chiabu.2018.07.018 30064076

[B21] Chou C. Bentler P. M. (1990). Model modification in covariance structure modeling: a comparison among likelihood ratio, lagrange multiplier, and wald tests. Multivar. Behav. Res. 25, 115–136. doi: 10.1207/s15327906mbr2501_13 26741976

[B22] Ciaffoni S. Rubini M. Moscatelli S. (2025). The unbearable weight of gender inequalities: development and validation of the social treatment and experiences of women (STEW) scale. Sex Roles 91:3. doi: 10.1007/s11199-024-01555-1

[B23] Clark R. Anderson N. B. Clark V. R. Williams D. R. (1999). Racism as a stressor for African Americans: a biopsychosocial model. Am. Psychol. 554, 805–816. doi: 10.1037/0003-066X.54.10.805 10540593

[B24] Collins J. W. Jr David, R. J. Symons R. Handler A. Wall S. N. Dwyer L. (2000). Low-income African-American mothers' perception of exposure to racial discrimination and infant birth weight. Epidemiology 1, 337–339. doi: 10.1097/00001648-200005000-00019 10784254

[B25] Compas B. E. Malcarne V. L. Fondacaro K. M. (1988). Coping with stressful events in older children and young adolescents. J. Consult. Clin. Psychol. 56, 405–411. doi: 10.1037/0022-006X.56.3.405 3397433

[B26] Crenshaw K. (1991). Mapping the margins: intersectionality, identity politics, and violence against women of color. Stanford Law Rev. 43, 1241–1299. doi: 10.2307/1229039

[B27] Crittenden P. M. Dallos R. (2009). All in the family: integrating attachment and family systems theories. Clinical Child Psychol. Psychiatry 14, 389–409. doi: 10.1177/1359104509104048 19515755

[B28] Dagan O. Sagi-Schwartz A. van IJzendoorn M. H. (2021). Attachment networks to multiple caregivers: an introduction to a special issue. New Direct. Child Adolesc. Dev. 2021, 5–7. doi: 10.1002/cad.20453 35174961

[B29] Dong L. (2024). Early attachment and socioemotional development of adolescence and adulthood: the mediating role of emotion regulation and self-esteem. SHS Web Conf. 193:2013. doi: 10.1051/shsconf/202419302013

[B30] Dotterer A. M. James A. (2018). Can parenting microprotections buffer against adolescents' experiences of racial discrimination? J. Youth Adolesc. 47, 38–50. doi: 10.1007/s10964-017-0773-6 29052120

[B31] Dunbar A. S. Leerkes E. M. Coard S. I. Supple A. J. Calkins S. (2017). An integrative conceptual model of parental racial/ethnic and emotion socialization and links to children's social-emotional development among African American families. Child Dev. Perspect. 11, 16–22. doi: 10.1111/cdep.12218

[B32] Eisenberg N. Cumberland A. Spinrad T. L. (1998). Parental socialization of emotion. Psychol. Inq. 9, 241–273. doi: 10.1207/s15327965pli0904_1 16865170 PMC1513625

[B33] Erikson E. H. (1968). Identity: Youth and Crisis. New York: W. W. Norton.

[B34] Eschenbeck H. Kohlmann C.-W. Lohaus A. (2007). Gender differences in coping strategies in children and adolescents. J. Individ. Differen. 28, 18–26. doi: 10.1027/1614-0001.28.1.18

[B35] Farah M. J. Betancourt L. Shera D. M. Savage J. H. Giannetta J. M. Brodsky N. L. . (2008). Environmental stimulation, parental nurturance and cognitive development in humans. Dev. Sci. 11, 793–801. doi: 10.1111/j.1467-7687.2008.00688.x 18810850

[B36] Farrell C. Slaughter V. Thai M. Mulvihill A. (2023). How we talk to kids: adults prefer different forms of language for children based on gender expression. Sex Roles 89, 119–134. doi: 10.1007/s11199-023-01393-7

[B37] Faul F. Erdfelder E. Lang A.-G. Buchner A. (2007). G^*^Power 3: a flexible statistical power analysis program for the social, behavioral, and biomedical sciences. Behav. Res. Met. 39, 175–191. doi: 10.3758/BF03193146 17695343

[B38] Ferguson G. M. Bornstein M. H. Pottinger A. M. (2012). Tridimensional acculturation and adaptation among Jamaican adolescent-mother dyads in the United States. Child Dev. 83, 1486–1493. doi: 10.1111/j.1467-8624.2012.01787.x 22966917 PMC3442943

[B39] Finley A. J. Baldwin C. L. Hebbring T. M. van Reekum C. M. Thayer J. F. Davidson R. J. . (2024). Differences in emotion expression, suppression, and cardiovascular consequences between Black and White Americans in the midlife in the United States (MIDUS) Study. Psychos. Med. 86, 748–757. doi: 10.1097/PSY.0000000000001348 39412291 PMC11560665

[B40] Folkman S. Lazarus R. S. Pimley S. Novacek J. (1987). Age differences in stress and coping processes. Psychol. Aging 2, 171–184. doi: 10.1037/0882-7974.2.2.171 3268206

[B41] Freitas A. L. Downey G. (1998). Resilience: a dynamic perspective. Int. J. Behav. Dev. 22, 263–285. doi: 10.1080/016502598384379

[B42] Gaylord-Harden N. K. Cunningham J. A. (2009). The impact of racial discrimination and coping strategies on internalizing symptoms in African American youth. J. Youth Adolesc. 38, 532–543. doi: 10.1007/s10964-008-9377-5 19636726

[B43] Gross J. J. John O. P. (2003). Individual differences in two emotion regulation processes: implications for affect, relationships, and well-being. J. Pers. Soc. Psychol. 85:348. doi: 10.1037/0022-3514.85.2.348 12916575

[B44] Gross J. J. Levenson R. W. (1993). Emotional suppression: physiology, self-report, and expressive behavior. J. Personal. Soc. Psychol. 64, 970–986. doi: 10.1037/0022-3514.64.6.970 8326473

[B45] Grossmann K. Grossmann K. E. Fremmer-Bombik E. Kindler H. Scheuerer-Englisch H. Zimmermann P. (2002). The uniqueness of the child-father attachment relationship: fathers' sensitive and challenging play as a pivotal variable in a 16-year longitudinal study. Soc. Dev. 11, 307–331. doi: 10.1111/1467-9507.00202

[B46] Hazan C. Shaver P. R. (1994). Attachment as an organizational framework for research on close relationships. Psychol. Inq. 5, 1–22. doi: 10.1207/s15327965pli0501_1

[B47] Henry J. S. Lambert S. F. Smith Bynum M. (2015). The protective role of maternal racial socialization for African American adolescents exposed to community violence. J. Family Psychol. J. Division Family Psychol. Am. Psychol. Assoc. 29, 548–557. doi: 10.1037/fam0000135 26374933

[B48] Hill S. A. (2001). Class, race, and gender dimensions of child rearing in African American families. J. Black Stud. 31, 494–508. doi: 10.1177/002193470103100407

[B49] Hu L. Bentler P. M. (1999). Cutoff criteria for fit indexes in covariance structure analysis: conventional criteria versus new alternatives. Struct. Eq. Model. Multidiscipl. J. 6, 1–55. doi: 10.1080/10705519909540118

[B50] Hughes D. Rodriguez J. Smith E. P. Johnson D. J. Stevenson H. C. Spicer P. (2006). Parents' ethnic-racial socialization practices: a review of research and directions for future study. Dev. Psychol. 42, 747–770. doi: 10.1037/0012-1649.42.5.747 16953684

[B51] Huguley J. P. Wang M. T. Vasquez A. C. Guo J. (2019). Parental ethnic-racial socialization practices and the construction of children of color's ethnic-racial identity: a research synthesis and meta-analysis. Psychol. Bull. 145, 437–458. doi: 10.1037/bul0000187 30896188

[B52] Ibe-Lamberts K. Ajibewa T. Onyeise D. McNeil-Young V. Nmezi N. A. Williams J. (2025). “I'm Black and i'm stressed”: an exploratory literature review on stress and coping mechanisms among African Americans, Afro-Caribbean, and African Immigrants in the USA. *J. Racial Ethn. Health Disparities* doi: 10.1007/s40615-025-02394-w. [Epub ahead of print]. 40146467

[B53] IBM Corp (2020). IBM SPSS Statistics for Windows (Version 27.0) [Computer software]. IBM Corp.

[B54] Ishak N. M. Yunus M. M. Iskandar I. P. (2010). Trust, communication and healthy parental attachment among Malaysian academically talented college students. Proced. Soc. Behav. Sci., 9, 1529–1536. doi: 10.1016/j.sbspro.2010.12.360

[B55] Islamiah N. Breinholst S. Walczak M. A. Esbjørn B. H. (2023). The role of fathers in children's emotion regulation development: a systematic review. Infant Child Dev. 32:e2397. doi: 10.1002/icd.2397

[B56] Jackson J. F. (1993). Multiple caregiving among African Americans and infant attachment: the need for an emic approach. Human Dev. 36, 87–102. doi: 10.1159/000277299

[B57] Jacob G. Faber S. C. Faber N. Bartlett A. Ouimet A. J. Williams M. T. (2023). A systematic review of black people coping with racism: approaches, analysis, and empowerment. Perspect. Psychol. Sci. 18, 392–415. doi: 10.1177/17456916221100509 36006823 PMC10018067

[B58] Jiménez A. Glover C. S. (2023). Racial discrimination distress and psychological well-being: the moderating role of ethnic-racial socialization in African American emerging adults. Emerg. Adult. 11, 639–653. doi: 10.1177/21676968231151981

[B59] Kerig P. K. Swanson J. A. Ward R. M. (2012). “Autonomy with connection: influences of parental psychological control on mutuality in emerging adults' close relationships,” in Adolescence and Beyond: Family Processes and Development, eds. Kerig P. K. Schulz M. S. and Hauser S. T. (Oxford: Oxford University Press), 134–153. doi: 10.1093/acprof:oso/9780199736546.003.0009

[B60] Kim H. K. McKenry P. C. (1998). Social networks and support: a comparison of African Americans, Asian Americans, Caucasians, and Hispanics. J. Comparat. Family Stud. 29, 313–334. doi: 10.3138/jcfs.29.2.313

[B61] Labella M. H. (2018). The sociocultural context of emotion socialization in African American families. Clin. Psychol. Rev. 59, 1–15. doi: 10.1016/j.cpr.2017.10.006 29150177

[B62] Lewis J. A. Mendenhall R. Harwood S. A. Huntt M. B. (2013). Coping with gendered racial microaggressions among black women college students. J. Afr. Am. Stud. 17, 51–73. doi: 10.1007/s12111-012-9219-0

[B63] Lewis-Coles M. E. L. Constantine M. G. (2006). Racism-related stress, Africultural coping, and religious problem-solving among African Americans. Cult. Divers. Ethn. Minor. Psychol. 12, 433–443. doi: 10.1037/1099-9809.12.3.433 16881748

[B64] Lindsey M. A. Brown D. R. Cunningham M. (2017). Boys do(n't) cry: addressing the unmet mental health needs of African American boys. Am. J. Orthopsychiatry 87, 377–383. doi: 10.1037/ort0000198 28691838

[B65] MacCallum R. C. Browne M. W. andamp; Sugawara H. M. (1996). Power analysis and determination of sample size for covariance structure modeling. Psychol. Met. 1, 130–145. doi: 10.1037/1082-989X.1.2.130

[B66] Marcia J. E. (1966). Development and validation of ego-identity status. J. Personal. Soc. Psychol. 3, 551–558. doi: 10.1037/h0023281 5939604

[B67] McEwen B. S. (1998). Protective and damaging effects of stress mediators. New Engl. J. Med. 338, 171–179. doi: 10.1056/NEJM199801153380307 9428819

[B68] Metzger I. W. Cooper S. M. Griffin C. B. Golden A. R. Opara I. Ritchwood T. D. (2020). Parenting profiles of academic and racial socialization: associations with academic engagement and academic self-beliefs of African American adolescents. J. School Psychol. 82, 36–48. doi: 10.1016/j.jsp.2020.07.001 32988462 PMC8219402

[B69] Morris A. S. Silk J. S. Steinberg L. Myers S. S. Robinson L. R. (2007). The role of the family context in the development of emotion regulation. Soc. Dev. 16, 361–388. doi: 10.1111/j.1467-9507.2007.00389.x 19756175 PMC2743505

[B70] Morsbach S. K. Prinz R. J. (2006). Understanding and Improving the Validity of Self-Report of Parenting. Clin. Child Family Psychol. Rev. 9, 1–21. doi: 10.1007/s10567-006-0001-5 16636897

[B71] Muthén B. Muthén L. (2017). “Mplus,” in Handbook of Item Response Theory, ed W. J. van der Linden (Boca Raton, FL: Chapman & Hall/CRC), 507–518.

[B72] Nadal K. L. (2011). The Racial and Ethnic Microaggressions Scale (REMS): construction, reliability, and validity. J. Counsel. Psychol. 58, 470–480. doi: 10.1037/a0025193 21875180

[B73] Nadal K. L. Griffin K. E. Wong Y. Hamit S. Rasmus M. (2014a). The impact of racial microaggressions on mental health: counseling implications for clients of color. J. Couns. Dev. 92, 57–66. doi: 10.1002/j.1556-6676.2014.00130.x

[B74] Nadal K. L. Wong Y. Griffin K. E. Davidoff K. Sriken J. (2014b). The adverse impact of racial microaggressions on college students' self-esteem. J. College Stud. Dev. 55, 461–474. doi: 10.1353/csd.2014.0051

[B75] Nelson S. C. Syed M. Tran A. G. T. T. Hu A. W. Lee R. M. (2018). Pathways to ethnic-racial identity development and psychological adjustment: the differential associations of cultural socialization by parents and peers. Dev. Psychol. 54, 2166–2180. doi: 10.1037/dev0000597 30265036

[B76] Pahl K. Way N. (2006). Longitudinal trajectories of ethnic identity among urban black and Latino adolescents. Child Dev. 77, 1403–1415. doi: 10.1111/j.1467-8624.2006.00943.x 16999807

[B77] Pederson D. R. Gleason K. E. Moran G. Bento S. (1998). Maternal attachment representations, maternal sensitivity, and the infant–mother attachment relationship. Dev. Psychol. 34, 925–933. doi: 10.1037/0012-1649.34.5.925 9779739

[B78] Perry N. S. Dieujuste N. Parsons A. Stanley S. M. Rhoades G. K. (2024). Racial discrimination and parenting perceptions among low-income Black couples. J. Fam. Psychol. doi: 10.1037/fam0001207. [Epub ahead of print]. 38421762

[B79] Pew Research Center (2024). Racial Discrimination Shapes how Black Americans View their Progress and U.S. Institutions. Washington, DC: Pew Research Center.

[B80] Pietromonaco P. R. Barrett L. F. (2000). The internal working models concept: what do we really know about the self in relation to others? Rev. Gen. Psychol. 4, 155–175. doi: 10.1037//1089-2680.4.2.155

[B81] Preacher K. J. Rucker D. D. Hayes A. F. (2007). Addressing moderated mediation hypotheses: theory, methods, and prescriptions. Multiv. Behav. Res. 42, 185–227. doi: 10.1080/00273170701341316 26821081

[B82] Reupert A. Straussner S. L. Weimand B. Maybery D. (2022). It Takes a Village to Raise a Child: Understanding and Expanding the Concept of the “Village”. Front. Public Health 10:756066. doi: 10.3389/fpubh.2022.756066 35372232 PMC8964422

[B83] Reynolds J. E. Gonzales-Backen M. A. (2017). Ethnic-racial socialization and the mental health of African Americans: a critical review. J. Family Theory Rev. 9, 182–200. doi: 10.1111/jftr.12192

[B84] Saleem F. T. Lambert S. F. (2016). Differential effects of racial socialization messages for African American adolescents: personal versus institutional racial discrimination. J. Child Family Stud. 25, 1385–1396. doi: 10.1007/s10826-015-0326-0

[B85] Salem D. A. Zimmerman M. A. Notaro P. C. (1998). Effects of family structure, family process, and father involvement on psychosocial outcomes among African American adolescents. Family Relat. Interdiscipl. J. Appl. Family Stud. 47, 331–341. doi: 10.2307/585264

[B86] Sanchez D. Adams W. N. Arango S. C. Flannigan A. E. (2018). Racial-ethnic microaggressions, coping strategies, and mental health in Asian American and Latinx American college students: a mediation model. J. Counsel. Psychol. 65, 214–225. doi: 10.1037/cou0000249 29543476 PMC10371219

[B87] Sandler I. Miles J. Cookston J. Braver S. (2008). Effects of father and mother parenting on children's mental health in high- and low-conflict divorces. Family Court Rev. 46, 282–296. doi: 10.1111/j.1744-1617.2008.00201.x

[B88] Satorra A. (2000). “Scaled and adjusted restricted tests in multi-sample analysis of moment structures,” in Innovations in Multivariate Statistical Analysis (New York: Springer), 233–247. doi: 10.1007/978-1-4615-4603-0_17

[B89] Seaton E. K. Upton R. Gilbert A. Volpe V. (2014). A moderated mediation model: racial discrimination, coping strategies, and racial identity among Black adolescents. Child Dev. 85, 882–890. doi: 10.1111/cdev.12122 23668685 PMC6673645

[B90] Segal D. L. Coolidge F. L. Mizuno H. (2007). Defense mechanism differences between younger and older adults: a cross-sectional investigation. Aging Mental Health 11, 415–422. doi: 10.1080/13607860600963588 17612805

[B91] Sellers R. M. Shelton J. N. (2003). The role of racial identity in perceived racial discrimination. J. Person. Soc. Psychol. 84, 1079–1092. doi: 10.1037/0022-3514.84.5.1079 12757150

[B92] Shaver P. R. Collins N. Clark C. L. (1996). “Attachment styles and internal working models of self and relationship partners,” in Knowledge Structures in Close Relationships: A Social Psychological Approach, eds Fletcher G. J. O. and Fitness J. (Mahwah, NJ: Erlbaum), 25–61.

[B93] Sheu H.-B. Sedlacek W. E. (2004). An exploratory study of help-seeking attitudes and coping strategies among college students by race and gender. Measure. Eval. Counsel. Dev. 37, 130–143. doi: 10.1080/07481756.2004.11909755

[B94] Shieh G. (2010). Sample size determination for confidence intervals of interaction effects in moderated multiple regression with continuous predictor and moderator variables. Behav. Res. Met. 42, 824–835. doi: 10.3758/BRM.42.3.824 20805605

[B95] Skinner E. Edge K. (1998). Reflections on coping and development across the lifespan. Int. J. Behav. Dev. 22, 357–366.

[B96] Spencer M. B. Dupree D. Hartmann T. (1997). A phenomenological variant of ecological systems theory (PVEST): a self-organization perspective in context. Dev. Psychopathol. 9, 817–833. doi: 10.1080/016502598384414 9449007

[B97] Sroufe L. A. (2005). Attachment and development: a prospective, longitudinal study from birth to adulthood. Attach. Human Dev. 7, 349–367. doi: 10.1080/14616730500365928 16332580

[B98] Staples R. Johnson L. B. (1993). Black Families at the Crossroads:Challenges and Prospects. Hoboken, NJ: John Wiley & Sons, Inc.

[B99] Stern J. A. Jones J. D. Nortey B. M. Lejuez C. W. Cassidy J. (2022). Pathways linking attachment and depressive symptoms for Black and White adolescents: do race and neighborhood racism matter? Attach. Human Dev. 24, 304–321. doi: 10.1080/14616734.2021.1976924 34528475 PMC8924014

[B100] Suárez-Orozco C. Motti-Stefanidi F. Marks A. Katsiaficas D. (2018). An integrative risk and resilience model for understanding the adaptation of immigrant-origin children and youth. Am. Psycholog. 73, 781–796. doi: 10.1037/amp0000265 30188166

[B101] Szczygieł D. Maruszewski T. (2015). Why expressive suppression does not pay? Cognitive costs of negative emotion suppression: the mediating role of subjective tense-arousal. Polish Psychol. Bull. 46, 336–349. doi: 10.1515/ppb-2015-0041

[B102] Tucker L. R. Lewis C. (1973). A reliability coefficient for maximum likelihood factor analysis. Psychometrika 38, 1–10. doi: 10.1007/BF02291170

[B103] Tyrell F. A. Wang Y. S. Eboigbe L. I. Skeeter B. D. (2024). A multisystem model for understanding stress and adaptation in ethnically and racially diverse youth. Dev. Psychopathol. 36, 2439–2451. doi: 10.1017/S0954579424000592 38506061

[B104] Utsey S. O. Adams E. P. Bolden M. (2000). Development and initial validation of the africultural coping systems inventory. J. Black Psychol. 26, 194–215. doi: 10.1177/0095798400026002005

[B105] Varner F. Holloway K. R. Scott L. E. (2021). The roles of gender and parenting in the relations between racial discrimination experiences and problem behaviors among African American adolescents. Res. Human Dev. 18, 256–273. doi: 10.1080/15427609.2021.2020583 35340406 PMC8953153

[B106] Wang M.-T. Henry D. A. Smith L. V. Huguley J. P. Guo J. (2020). Parental ethnic-racial socialization practices and children of color's psychosocial and behavioral adjustment: a systematic review and meta-analysis. Am. Psychol. 75, 1–22. doi: 10.1037/amp0000464 31058521

[B107] Wang Y. Benner A. D. (2016). Cultural socialization across contexts: family–peer congruence and adolescent well-being. J. Youth Adolesc. 45, 594–611. doi: 10.1007/s10964-016-0426-1 26809337 PMC5353358

[B108] Wiggins E. R. Brisson J. M. Lavner J. A. Ehrlich K. B. (2023). The benefits of nurturant-involved parenting for children's internalizing symptoms and cardiometabolic health in high-risk contexts. Dev. Psychopathol. 35, 2420–2429. doi: 10.1017/S0954579423000652 37386849 PMC11228812

[B109] Xing S. Liang X. Yue J. Wang Z. (2016). Multiple attachment relationships and the impacts on children's socio-emotional development under the background of grandmother co-parenting. Acta Psychol. Sinica 48, 518–528. doi: 10.3724/SP.J.1041.2016.00518

[B110] Yan J. Jelsma E. Wang Y. Zhang Y. Zhao Z. Cham H. . (2024). Racial–ethnic discrimination and early adolescents' behavioral problems: the protective role of parental warmth. J. Am. Acad. Child Adolesc. Psychiatry 64, 249–261. doi: 10.1016/j.jaac.2024.03.020 38718977 PMC11538377

[B111] Zimmermann P. Iwanski A. (2014). Emotion regulation from early adolescence to emerging adulthood and middle adulthood: age differences, gender differences, and emotion-specific developmental variations. Int. J. Behav. Dev. 38, 182–194. doi: 10.1177/0165025413515405

