# Supplementary material for: Weathering the storm: how attachment and gender influence coping with racial discrimination
Source: Front Psychol. 2026 Mar 13;17:1709353. doi: 10.3389/fpsyg.2026.1709353 (PMC13021443; doi:10.3389/fpsyg.2026.1709353)
Supplement: Supplementary file 1 [file Table_1.docx]

Supplementary Materials

The primary path models above were re-estimated with both outcomes (collective coping and expressive suppression) in the same model. All paths, constraints, and model specifications remained the same as in the original analyses. The results were not consistent with the main findings reported in the manuscript. More specifically, none of the interaction effects were significant, indicating that we did not have sufficient power (i.e., requires a larger sample size) to detect these effects.

**Supplemental Aim 1: The Direct Effects between REMS and Coping Strategies**

To test the direct effect of racial ethnic microaggressions on both collective coping and expressive suppression, an additional model was estimated (see Table S1). The model fit for collective coping and expressive suppression was acceptable, *X^2^* = 4.963, *df* = 7, *p* = .6644; *RMSEA* = .000, 90% [.000, .065]; *CFI/ TLI* = 1.000/1.000; *SRMR* = .025. REMS was not associated significantly with collective coping, *t* = 1.360, *p* = .174, as well as maternal education, *t* = -0.363, *p* = .717, and immigration status, *t* = -0.663, *p* = .507. However, age, *t* = 2.595, *p* = .009, and female gender, *t* = 1.981, *p* = .048, were significant and positively associated with collective coping. REMS was also not associated significantly with expressive suppression, *t* = 1.347, *p* = .175. Female gender (*t* = 3.320, p = .001) was negatively associated with expressive suppression, but age (*t* = -0.418, *p* = .676), maternal education (*t* = -1.6521 p = .099), and immigration status (*t* = .330, *p* = .741) were not associated significantly with expression suppression.

**Supplemental Aim 2: The Moderating Effects of Parental Attachment on REMS and Coping**

Additional path analyses were conducted to assess the interaction effects of mother–youth and father–youth attachment on the associations between REMS with both coping strategies.

***REMS, MYA, and Coping***

The first path analysis evaluated the moderating effect on mother–youth on the association between REMS and both collective coping and expressive suppression (see Table S2). The model fit for coping was not acceptable, *X^2^* = 32.067, *df* = 11, *p* = 0.007; *RMSEA* = .091, 90% [.055, .129]; *CFI/ TLI* = .383 /.158; *SRMR* = .048. In contrast to the direct effects models, after controlling for the covariates and adding mother–youth attachment to the model, REMS became positively associated with collective coping, t = 2.047, p = .041. Mother-youth attachment, t = .721, p = .471, was not associated with collective coping. Additionally, the interaction between mother–youth attachment and REMS was not significant in predicting collective coping, t = .500, p = .617. Similarly, REMS showed a positive significant association with expressive suppression in this model, t = 2.122, p = .034. Mother-youth attachment was not associated with expressive suppression, t = .735, p = .462. Additionally, the interaction between mother–youth attachment and REMS was not significant when predicting expressive suppression, t = .502, p = .615.

***REMS, FYA, and Coping***

The second path analysis evaluated the moderating effect of father–youth attachment on the association between REMS and both collective coping and expressive suppression (see Table S2). . The model fit for coping was not acceptable *X^2^* = 20.057, *df* = 10, *p* = 0.0287; *RMSEA* = .066, 90% [.021, .108]; *CFI/ TLI* = .651 /.476; *SRMR* = .039. Similarly to the direct model, REMS was not associated with collective coping, t = 1.579, p = .114, after controlling for the covariates and father–youth attachment (see Table S2). Additionally, father–youth attachment was not associated with collective coping, t = .942, p = .346. There was no significant interaction between REMS and father–youth attachment on collective coping, t = -.973, p = .331. Additionally, REMS showed no significant association with expressive suppression, t = 1.579, p = .114 in this model. Father-youth attachment was not significantly related to expressive suppression, t = .942, p = .346. The interaction between father–youth attachment and REMS was also not significant in predicting expressive suppression, t = -.973, p = .331.

**Supplemental Aim 3: The Moderating Effects of Immigration Status and Attachment on REMS and Coping**

Path models were estimated to evaluate the moderating effect of immigration status on the associations among REMS, mother–youth attachment or father–youth attachment and collective coping or expressive suppression. A multigroup analysis using the WALD test was utilized to examine whether interaction parameter estimates differed across groups for immigration status. Similar to the other models, the paths, constraints, and model specifications remained the same as those in the original analyses.

Across all models, the Wald test analyses [Wald χ²(1) = .152-3.713, *ps =*.0540-.6963] revealed no significant differences across immigration status.

Supplemental Tables

**Table S1**

*The Direct Effects of Racial Ethnic Microaggressions on both Collective Coping and Expressive Suppression in Black emerging adults.*

| Predictors | Collective Coping | | Expressive Suppression | |
| --- | --- | --- | --- | --- |
|  | β | *SE* | β | *SE* |
| REMS | .10 | .07 | .08 | .06 |
| Age | .18* | .07 | –.03 | .07 |
| Gender | .13* | .07 | –.22** | .07 |
| Maternal Education | –.03 | .07 | –.11^☩^ | .07 |
| Immigration Status | –.05 | .07 | .02 | .07 |
| R^2^ | .07* | .04 | .05* | .03 |

*Note.* REMS = Racial Ethnic Microaggressions; Gender was coded as 0 = male, 1 = female; Immigration status was coded as 0 = no immigrant background, 1 = immigrant background; β *=* standardized regression coefficient*; SE* = standard error

* *p* < .05. ** *p* < .01. *** *p* < .001. ^☩^ marginally significant effect.

**Table S2**

*The Moderated Effects of Mother-Youth Attachment and Father-Youth Attachment on the Associations of Racial-Ethnic Microaggressions on both Collective Coping and Expressive Suppression*

| Predictors | Collective Coping | | Expressive Suppression | |
| --- | --- | --- | --- | --- |
|  | β | *SE* | β | *SE* |
| Mother-Youth Attachment | | | | |
| REMS | .10* | .05 | .10* | .05 |
| MYA | .04 | .05 | .04 | .05 |
| REMS * MYA | .02 | .05 | .02 | .05 |
| Age | .18* | .07 | –.03 | .07 |
| Gender | .13^☩^ | .07 | –.21* | .07 |
| Maternal Education | –.04 | .07 | –.12^☩^ | .07 |
| Immigration Status | –.04 | .07 | –.02 | .07 |
| R^2^ | .05 | .03 | .08** | .03 |
| Father-Youth Attachment | | | | |
| REMS | .08 | .05 | .08 | .05 |
| FYA | .04 | .05 | .05 | .05 |
| REMS * FYA | –.04 | .04 | –.04 | .05 |
| Age | .18* | .07 | –.03 | .07 |
| Gender | .14* | .07 | –.21* | .07 |
| Maternal Education | –.02 | .07 | –.11^☩^ | .07 |
| Immigration Status | –.04 | .07 | .03 | .07 |
| R^2^ | .05* | .03 | .08** | .04 |

*Note.* REMS = Racial Ethnic Microaggressions; MYA= Mother-Youth Attachment; FYA= Father-Youth Attachment; Gender was coded as 0 = male, 1 = female; Immigration status was coded as 0 = no immigrant background, 1 = immigrant background; *SE* = standard error; R^2^ = R- Squared.

* *p* < .05. ** *p* < .01. *** *p* < .001. ^☩^ marginally significant effect.
